# Targeted disruption of *Rab10* causes early embryonic lethality

**DOI:** 10.1007/s13238-015-0150-8

**Published:** 2015-04-10

**Authors:** Pingping Lv, Yi Sheng, Zhenao Zhao, Wei Zhao, Lusheng Gu, Tao Xu, Eli Song

**Affiliations:** 1 National Laboratory of Biomacromolecules, Institute of Biophysics, Chinese Academy of Sciences, Beijing, 100101 China; 2 University of Chinese Academy of Sciences, Beijing, 100049 China; 3 College of Life Science and Technology, Huazhong University of Science and Technology, Wuhan, 430074 China; 4 State Key Laboratory of Reproductive Biology, Institute of Zoology, Chinese Academy of Sciences, Beijing, 100101 China

**Dear Editor,**

Intracellular trafficking is a basis of cellular activities, including cell migration, immune response, and development (Lebreton et al., 2003; Lučin et al., 2014; Ulrich and Heisenberg, 2009). In healthy tissue, intracellular trafficking is highly regulated, controlling the form and function of cells (Furthauer and Gonzalez-Gaitan, 2009). Work carried out in animals and plants highlights that regulated intracellular trafficking is important for the development of multicellular organisms (Fürthauer and González-Gaitán, 2009; Kolotuev et al., 2009; Richter et al., 2009). As a family of Ras-related small GTPase, Rabs function as coordinators for diverse aspects of intracellular trafficking including vesicle budding and formation, cargo sorting and transport to target membranes, and recruitment of key molecules (Stenmark, 2009; Zerial and McBride, 2001).

Rab10, widely distributed in intracellular membranes, is highly conserved from *Caenorhabditis elegans* (*C. elegans*) to humans. In the *C.* *elegans* intestine, Rab10 coordinated the transport of clathrin-independent cargo between early endosomes and recycling endosomes (Chen et al., 2006; Chen et al., 2014). Recent work in Drosophila follicle cells suggested that Rab10 is related to polarized basement membrane secretion during organ morphogenesis (Lerner et al., 2013). In mammalian systems, Rab10 has been implicated in mediating membrane targeting of plasmalemmal precursor vesicles (PPVs) during axon development (Xu et al., 2014). In addition, a large body of evidence has identify Rab10 as an essential component in basolateral recycling in Madin-Darby canine kidney (MDCK) cells and in insulin stimulated GLUT4 recycling in adipocytes (Babbey et al., 2006; Chen et al., 2012). These accumulating results strongly suggest that Rab10 is crucial and has extensive functions in cell biological processes; however, the physiological functions of Rab10 *in vivo* remain unclear.

**Figure 1:**
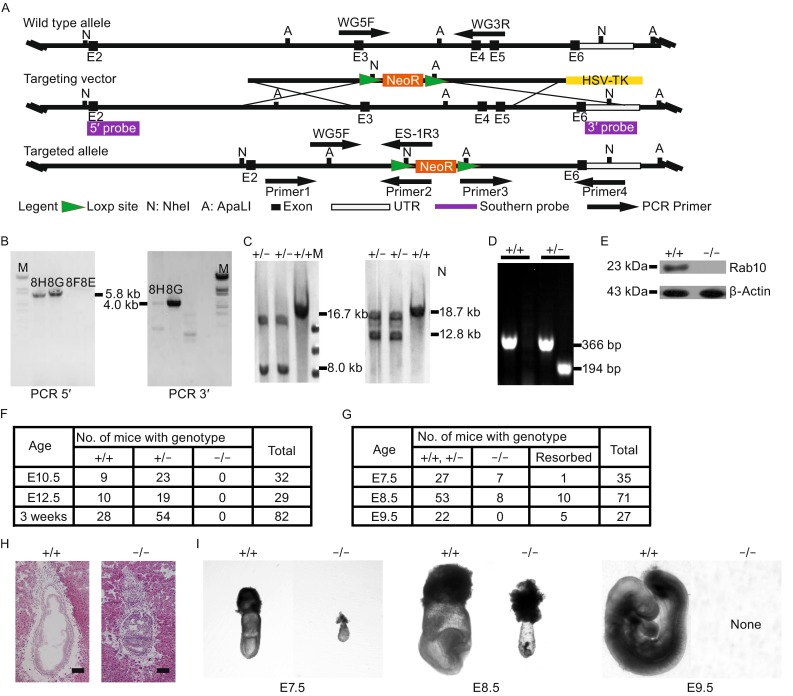
**Rab10 deficiency causes early embryonic lethality**. (A) Schematic representation of the *Rab10* locus, the targeting vector, and the targeted locus. The black boxes represent exon 2–6 of *Rab10* gene. Arrows show the primers used for PCR analysis. (B) Left: PCR analysis to detect 5′ recombination in transfected ES cells with primers 1 and 2. Right: PCR analysis to detect 3′ recombination with primers 3 and 4. M: size marker. (C) Southern blot analysis to identify correctly targeted *Rab10* ^+/−^ mice. A segment of 8.0 kb at the 5′ terminal end and a segment of 12.8 kb at the 3′ terminal end were detected in *Rab10* ^+/−^ mice. (D) Genotyping of *Rab10* ^+/+^ and *Rab10* ^+/−^ mice by PCR analysis. (E) Rab10 level was analyzed by Western blot in *Rab10* ^+/+^ and *Rab10* ^−/−^ embryos. (F) Genotypes of progeny aged E10.5, E12.5 and 3 weeks after birth from *Rab10* heterozygote intercrosses. (G)* Rab10* ^−/−^ embryos from intercrosses of heterozygous littermates recovered at E7.5. (H) Histological analysis of E7.5 embryos developing in the uterus. Typical pictures of wild-type and *Rab10* knockout embryos are displayed in the left and right panels, respectively. Scale bar = 100 μm. (I) Appearance of Rab10-deficient embryos from E7.5 to E8.5 and totally resorbed at E9.5

In this study, we attempted to generate Rab10-deficient mice via homologous recombination in mouse embryonic stem cells to determine the contribution of Rab10 *in vivo*. Mouse *Rab10* is located on chromosome 12 and is composed of 6 exons with the translational start codon ATG in exon 1. In our targeting strategy, we constructed a plasmid that replaced exons 3, 4, and 5 with a neomycin resistance gene cassette resulting in a frame shift in exon 6 (Fig. 1A).

The targeting plasmid was transfected into ES cells by electroporation. After screening with G418, positive ES clones were identified via PCR to amplify a 5.8 kb long arm and a 4.0 kb short arm (Fig. 1B). Four out of 102 cell clones displayed the desirable target, approximately 3.9%. After confirmation using Southern blot analysis, the targeted ES clones were injected into C57BL/6 blastocysts to generate chimeric mice which were backcrossed with C57BL/6 mice to produce heterozygous *Rab10* ^+/−^ mice. *Rab10* ^+/−^ mice were sequentially backcrossed with C57BL/6 for more than 10 generations to purify their background. Then *Rab10* ^+/−^ mice were confirmed by Southern blot (Fig. 1C) and the offspring’s genotypes were detected by PCR using genomic tail DNA as a template (Fig. 1D). Heterozygous *Rab10* ^+/−^ mice displayed no obvious abnormality in weight, diet or fertility during a 12-month observation period.

To generate Rab10-null mice, heterozygous mutants were intercrossed. Three weeks after birth, the offspring’s genotypes were identified. Although *Rab10* ^+/−^ parents showed no obvious abnormality, none of the 82 offspring were found to be homozygous mutants (*Rab10* ^−/−^) (Fig. 1F) and no increased neonatal mortality was observed in the initial 3 weeks after birth. The ratio between wild-type and heterozygote mice was 0.52, in accordance with Mendel’s law. These results suggest that one functional *Rab10* allele is enough for murine embryo development and adults’ survival, but that a double mutant leads to embryonic lethality.

To assess the period of developmental failure, embryos from heterozygote hybridization at embryonic day 12.5 (E12.5), 10.5, and 9.5 were collected and genotyped by PCR. As shown in Fig. 1F and 1G, none of the *Rab10* ^−/−^ embryos was found after E9.5, suggesting that the time of embryonic lethality was before E9.5. Therefore, we continued to analyze earlier embryos at E7.5 and E8.5. In total, out of 35 E7.5 embryos, 7 (20.0%) were homozygous mutants and 1 (2.9%) was a resorbed embryo; out of 71 E8.5 embryos, 8 (11.3%) were homozygous mutants and 10 (14.1%) were resorbed embryos (Fig. 1G). To further confirm these results, E7.5 embryos were collected and analyzed by Western blot with anti-Rab10 antibody. As shown in Fig. 1E, Rab10 protein was totally deleted in Rab10-null mice. *Rab10* ^−/−^ mutants showed obvious differences from wild-type embryos (Fig. 1I). Mutant embryos at E7.5 were much smaller and the development of the three layers (endoderm, mesoderm, and ectoderm) was largely arrested. Hematoxylin and eosin staining of histological sections of E7.5 are shown in Fig. 1H. Extraembryonic ectoderm of *Rab10* knockout embryos was not well organized and primitive streak formation was not observed in the embryonic region. The ectoplacental cavity, exocoelomic cavity, and amniotic cavity were abnormal. At E8.5, the embryos had degenerated much more seriously, with very little to almost no identifiable structure. Along with embryonic degeneration, the yolk sac was greatly degraded. Consequently, the abnormal embryos were totally absorbed at E9.5. Taken together, these data demonstrate that the development of *Rab10* ^−/−^ embryos is defective approximately at E7.5 and the mutant embryos are totally resorbed at E9.5.

**Figure 2:**
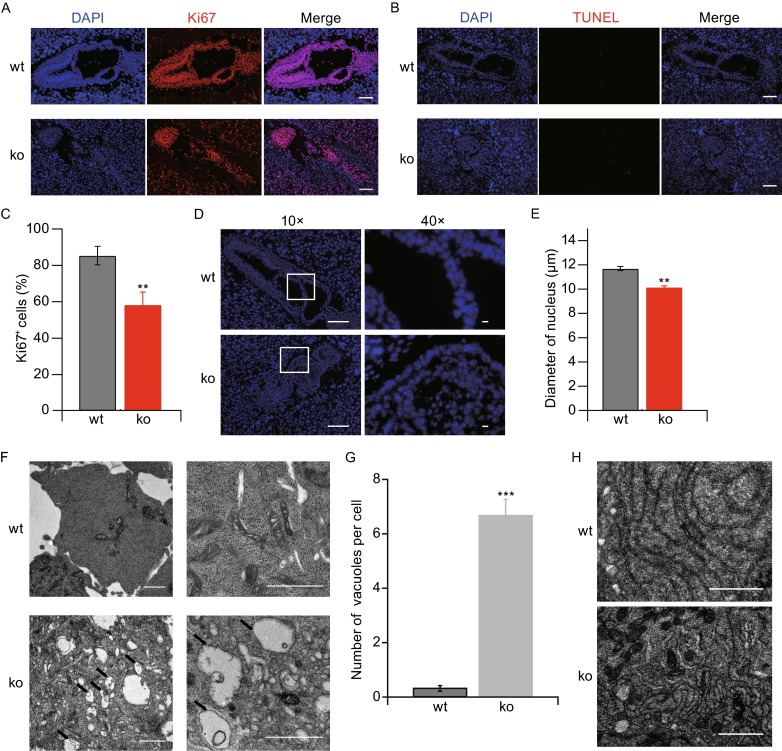
**E7.5** ***Rab10*** ^−/−^ **embryos are deficient in cell proliferation and show small nucleus, accumulated extensive vacuoles and hyperplasia ER**. (A) Cell proliferation was assessed using Ki67 staining in E7.5 embryos from heterozygous intercrosses, scale bar = 100 μm. (B) Rab10-deficient embryos did not undergo apoptosis. A TUNEL assay was performed for apoptosis detection using an *in situ* cell death detection kit (Beyotime). (C) Quantification of Ki67-positive cells in wild-type and mutant embryos. Values are shown as the average ± standard deviation, and the *P*-value was determined using a *t*-test. (D) Nuclear staining with DAPI of embryos at E7.5. The left panel, scale bar = 100 μm; the right panel, scale bar = 10 μm. (E) The nuclear size of *Rab10* ^−/−^ embryos was smaller. The images were analyzed using an image processing software to quantify the size of nuclei in wild-type and *Rab10* ^−/−^ embryos. (F) The ultrastructure of isolated E7.5 embryos was stained and visualized using electron microscopy. Black arrowheads indicate vacuoles. Scale bar = 1 μm. (G) Quantification of vacuoles in wild-type and mutant embryos. Values are expressed as the average ± standard deviation and the *P*-value was determined by a *t*-test. (H) The ER exhibited hyperplasia in *Rab10* ^−/−^ embryos. Total embryonic cells were analyzed in A and B; In (D), 4–5 parts of every embryo were randomly selected to calculate the size of nucleus; For (F) and (H), the cells were from epiblast

Based on our observation of E7.5 developmental failure above, we used an immunofluorescent assay with Ki67 (Abcam) and 4’,6-diamidino-2-phenylindole (DAPI) staining to determine whether defective development of *Rab10* ^−/−^ embryos was due to the failure of cell proliferation. The embryos were separated at E7.5 after fertilization to obtain 10 μm frozen sections for immunofluorescence. As shown in Fig. 2A and 2C, approximately 89% of the cells were Ki67-positive in wild-type embryos; however, only 60% of the cells were Ki67-positive in mutant embryos, suggesting a dramatic reduction in cellular proliferation.

None of the Rab10-null embryos grew after E7.5, and many of the cells were proliferative-inactive. To determine the reasons underlying the massive cell death in mutant conceptuses and eventual embryo destruction, terminal deoxynucleotidyl transferase-mediated dUTP-biotin nick end labeling (TUNEL) staining was used to detect the ratio of apoptotic cells in wild-type and *Rab10* ^−/−^ embryos (Fig. 2B). Embryonic frozen sections were stained with DAPI to label cell location and fluorescence microscopy was used to record 405 nm and 561 nm at the same time. Statistical analysis revealed that few TUNEL-positive cells were detected not only in wild-type embryos but also in *Rab10* ^−/−^ littermates, suggesting that Rab10-null embryos did not undergo significant apoptosis at E7.5.

When we analyzed the size of nucleus, however, we noticed that the nuclei diameter in the *Rab10* ^−/−^ mutants was reduced by 13% compared with that of the wild-type embryos. The diameter of the nuclei in wild-type embryos was 11.9 μm, whereas in Rab10-null mice, the diameter was 10.3 μm (Fig. 2D and 2E). Because of the critical role of the nucleus during cellular activities such as cell division, mRNA transcription and protein expression, the variation of nuclear size may be an indication of other significant cellular changes.

Because of the diminished number of cells and the decreased size of the nucleus in *Rab10* ^−/−^ embryos, we hypothesized that morphological changes in subcellular structures may be present in the *Rab10* mutants. It has been reported that in *C. elegance*, *Rab10* mutation caused enlarged vacuoles of early endosome origin by blocking export from the basolateral early endosomes (Chen et al., 2006). Because the *Rab10* gene is highly conserved between different species, we suspected that *Rab10* ^−/−^ cells may show a similar phenotype. We analyzed histological sections using immunofluorescence to label different organelles followed by confocal microscopy; however, structural details could not be observed clearly (data not shown) because the large scale of the nucleus occupied most of the cytoplasmic space and the resolution was limited. We then used transmission electron microscopy to study the ultrastructure in *Rab10* ^−/−^ samples. As shown in Fig. 2F, the cytoplasm was very dense and full of ribosomes in wild-type cells, which reflected the high synthetic activities of proteins. On the contrary, in *Rab10* ^−/−^ cells, many vacuoles were accumulated in the cytoplasm (Fig. 2F and 2G). The size of these vacuoles varied from 400 nm to 1600 nm. Moreover, the ER displayed hyperplasia compared with wild-type embryos (Fig. 2H). Considering these results, loss of Rab10 function results in abnormal endosomes and ER hyperplasia, likely disturbing the balance of membrane trafficking and ultimately leading to the death of the embryos.

In summary, we presented the study of Rab10 function in conventional knockout mice and demonstrated that Rab10 executes critical and non-redundant functions in the early development of mammalian embryos. First, no *Rab10* ^−/−^ mice or embryos were found after E9.5, and approximately 14.1% of embryos at E8.5 were completely resorbed. At E7.5, all of the abnormal embryos were *Rab10* ^−/−^. Second, the dramatic reduction of Ki67 positive cells in Rab10-null embryos, followed by cell death, suggested that a defect in cellular proliferation underlies the developmental failure.

Rab10 has been reported to localize in many organelles, such as ER, golgi, early endosome, recycling endosome, and mediates membrane trafficking within the cell, which counterbalances secretion and permits complex interplay between cells and their environment. Although the Rab10-deficient embryos’ growth ceased at E7.5 followed by resorption at E8.5, massive cell death did not result from apoptosis (Fig. 2B). RAB10 deficiency results in the accumulation of abnormally abundant RAB-5-positive vacuoles and it is regarded as a key regulator of endocytic recycling in polarized epithelial cells of the *C. elegans* intestine (Chen et al., 2006). In this study, we successfully observed this phenomenon in *Rab10* mutant embryos. Vacuoles of different sizes, presumably early endosomes, were accumulated in the cytoplasm of Rab10-defective cells, which indicated the endosomal trafficking was largely defected in *Rab10* ^−/−^ cells. The eukaryotic endosomal trafficking is essential for the internalization and trafficking of macromolecules, fluid, membranes, and membrane proteins. The disruption of endosomal trafficking that leads to a cellular dysfunction may be responsible for the death of *Rab10* ^−/−^ embryos.

In the development of mouse embryos, gastrulation is initiated in the epiblast at about E6.5 and three germ layers are well established at around E7.5. At E8.0, somites first appear and heart starts to form. Given that the developmental disruption of Rab10-null embryos occurred at approximately E7.5, we suspected Rab10 should function earlier before E7.5 in the development of mice embryos. However, the exact mechanism about why Rab10 deficiency results in early embryonic cell death and tissue abnormalities needs to be further characterized.

This work is the first attempt to access the physiological function of Rab10 protein using a *Rab10* conventional knockout mouse model. Here we clearly demonstrate that Rab10 is essential for early embryogenesis. However, as the *Rab10* mutation in mice was difficult to study due to the severity of the phenotype, conditional knockout strategy is required for further clarification of Rab10’s function. As *Rab10* is expressed in many tissues, including lymphoid organs, liver, testis, ovary, etc. (BioGPS), tissue-specific knockout mouse models are needed to further define its physiological functions at different tissues.
